# Evaluation of controllers for augmentative hip exoskeletons and their effects on metabolic cost of walking: explicit versus implicit synchronization

**DOI:** 10.3389/fbioe.2024.1324587

**Published:** 2024-03-12

**Authors:** Ali Reza Manzoori, Davide Malatesta, Julia Primavesi, Auke Ijspeert, Mohamed Bouri

**Affiliations:** ^1^ Biorobotics Laboratory, EPFL, Lausanne, Switzerland; ^2^ Institute of Sport Sciences, University of Lausanne (UNIL), Lausanne, Switzerland; ^3^ Translational Neural Engineering Laboratory, EPFL, Lausanne, Switzerland

**Keywords:** exoskeleton control, hip exoskeleton, gait assistance, lower-limb exoskeleton, robotic exoskeleton, metabolic cost

## Abstract

**Background:** Efficient gait assistance by augmentative exoskeletons depends on reliable control strategies. While numerous control methods and their effects on the metabolic cost of walking have been explored in the literature, the use of different exoskeletons and dissimilar protocols limit direct comparisons. In this article, we present and compare two controllers for hip exoskeletons with different synchronization paradigms.

**Methods:** The implicit-synchronization-based approach, termed the Simple Reflex Controller (SRC), determines the assistance as a function of the relative loading of the feet, resulting in an emerging torque profile continuously assisting extension during stance and flexion during swing. On the other hand, the Hip-Phase-based Torque profile controller (HPT) uses explicit synchronization and estimates the gait cycle percentage based on the hip angle, applying a predefined torque profile consisting of two shorter bursts of assistance during stance and swing. We tested the controllers with 23 naïve healthy participants walking on a treadmill at 4 km ⋅ h^−1^, without any substantial familiarization.

**Results:** Both controllers significantly reduced the metabolic rate compared to walking with the exoskeleton in passive mode, by 18.0% (SRC, *p* < 0.001) and 11.6% (HPT, *p* < 0.001). However, only the SRC led to a significant reduction compared to walking without the exoskeleton (8.8%, *p* = 0.004). The SRC also provided more mechanical power and led to bigger changes in the hip joint kinematics and walking cadence. Our analysis of mechanical powers based on a whole-body analysis suggested a reduce in ankle push-off under this controller. There was a strong correlation (Pearson’s *r* = 0.778, *p* < 0.001) between the metabolic savings achieved by each participant with the two controllers.

**Conclusion:** The extended assistance duration provided by the implicitly synchronized SRC enabled greater metabolic reductions compared to the more targeted assistance of the explicitly synchronized HPT. Despite the different assistance profiles and metabolic outcomes, the correlation between the metabolic reductions with the two controllers suggests a difference in individual responsiveness to assistance, prompting more investigations to explore the person-specific factors affecting assistance receptivity.

## 1 Introduction

One of the envisioned applications of lower-limb exoskeletons and exosuits which has seen many advances in the last decade is partial gait assistance for human augmentation (Sawicki et al., 2020). To this end, the assistance replaces part of the energy expended by the human muscles to reduce the effort and increase the endurance, or to supplement the muscle forces, while keeping the user in control. The potential users for these types of assistive devices range from completely able-bodied individuals (Mooney et al., 2014; Ding et al., 2016) to elderly persons (Lee et al., 2017a; Seo et al., 2017; Baud et al., 2018) and people with minor to moderate impairments who remain ambulatory (Awad et al., 2017; Ortlieb et al., 2017; Pour Aji Bishe et al., 2022). Single-joint devices (i.e., those assisting only one joint per leg) are more appealing for augmentation, mostly due to lighter weight, less intrusive structure, and also easier adaptation of human users to single-joint assistance (Young and Ferris, 2017; Franks et al., 2021). Hip and ankle joints are usually targeted, as more than 70% of the total positive power is provided by them across a range of different walking conditions (Nuckols et al., 2020). A clear advantage of targeting the hip joint is the more proximal placement of the device, which reduces the burden of the added mass on the user (Browning et al., 2007), and facilitates wearability and integration with the human body. Furthermore, generating forces using the hip muscles is less energetically efficient compared to the ankle (Umberger and Rubenson, 2011), which indicates more potential for promoting energy economy in gait through assistance.

In fact, a common and widely accepted metric for evaluating the overall performance of the augmentative exoskeletons is the metabolic cost of walking (Pinto-Fernandez et al., 2020), which is an objective measure of the energy expended by the user. The premise is that the more synchronized and appropriate the assistance is, the more likely it is that the users will reduce their muscular effort and allow it to be replaced by the assistance provided by the exoskeleton. Such a coordinated operation requires the exoskeleton to have a good estimation of the user’s intended movement. Temporal differences on the order of tens of milliseconds have been shown to have significant effects on the quality of assistance (Ding et al., 2016; Lee et al., 2017b). This makes the temporal coordination and synchronization a challenging task.

Control of the assistive device is pivotal to this challenge, since it is in charge of mapping the sensory input to the provided assistance. Many different control strategies have been introduced in the literature (Baud et al., 2021), showcasing a spectrum of characteristics, from precise torque profile adjustments for different tasks to adaptability for accommodating diverse gait patterns. In terms of temporal coordination of the assistance with the user, various approaches have been taken, as reviewed and classified by Lora-Millan et al. (2022). Broadly speaking, some algorithms rely on explicit synchronization either through the calculation of the continuous gait phase (referred to as percent gait cycle (%GC)), or by detection of discrete gait phases. In other methods such as proportional EMG control, the synchronization emerges organically from the sensory signals and the way they are mapped to the assistance. Instead of explicitly calculating the gait phase, the methods in this category leverage sensory data and the implicit phase-related information in them to determine the appropriate assistive action in each moment.

Controllers with implicit synchronization do not directly impose a predefined torque profile as a function of progression in the gait cycle, but determine the torque from the sensory signals either according to a defined map, or using model-based calculations. The most common case in the first subcategory is proportional myoelectric control (Ferris et al., 2006; He and Kiguchi, 2007; Grazi et al., 2018) where the assistance is determined from the muscle activation signals. Other approaches use kinematic signals as inputs, usually based on a desired trajectory linking the kinematics of the different joints (Vallery et al., 2007; Martínez et al., 2019). In the second subcategory, models based on the mechanics of walking (Lv and Gregg, 2018; Sharbafi et al., 2018; Fang and Lerner, 2021) or inspired by the human neuromuscular system and muscle-reflex models (Dzeladini et al., 2016; Shafer et al., 2021; Durandau et al., 2022) have been used to calculate the required assistance torque. While refining the details of the torque profile is generally not straightforward in these methods, they are typically more adaptive across different gaits compared to the strategies with explicit synchronization. However, these advantages may come at the cost of more complex formulations or the need for more intricate sensing.

In our laboratory, a control strategy has been developed with the aim of leveraging the advantages of implicit synchronization while utilizing minimal sensory information. The idea behind this strategy, titled Simple Reflex Controller (SRC), is to use a minimal reflex-like mapping to provide hip extension assistance during the stance phase and hip flexion assistance during swing (Baud, 2020). This strategy only requires approximate ground reaction force information, and contrary to most reflex-based methods, avoids reliance on event detection and discrete state transitions to increase the robustness and versatility of the controller, while removing the need for tedious tuning procedures. However, the performance of this controller in terms of effort reduction has not been investigated so far.

On the other hand, explicit synchronization allows for fine-tuning of the continuous torque profile or torque patterns in different phases of the gait cycle. The drawback is that the approaches based on explicit synchronization often require predefined torque profiles or trajectories for different gaits, and tend to be sensitive to misdetections of the gait phase and errors in the estimation of the %GC. These characteristics reduce the adaptability and robustness of the controllers. Explicit synchronization methods can be time-based or state-based. In time-based methods, the %GC is calculated by normalizing the elapsed time since heel-strike over the estimated duration of the gait cycle (Lewis and Ferris, 2011). Methods in this subcategory work well in steady and repetitive gaits such as treadmill walking, and are mostly used for proof-of-concept studies or investigating the human-exoskeleton interaction (Young et al., 2017; Kang et al., 2019). For more realistic implementations, state-based estimation methods which use the state variables of the human-exoskeleton system to extract gait phase information may be more appropriate. Examples include adaptive frequency oscillators that estimate the gait frequency (Ronsse et al., 2011; Yan et al., 2015), machine-learning-based methods that directly estimate the %GC from the sensory signals (Kang et al., 2021), or %GC estimation based on a special class of state variables termed “phase variables” (Quintero et al., 2017).

A phase variable is a state variable which increases monotonically over each gait cycle, and therefore can be used to parameterize the %GC (Gregg et al., 2014). In addition to being time-independent, using phase variables can potentially offer better synchronization in non-steady gait and even during perturbations (Villarreal and Gregg, 2016), as the instantaneous gait phase information is assumed to be directly encoded in the phase variable, thus eliminating the need for convergence over several gait cycles. These methods have mostly been explored in previous works focused on estimation only (Quintero et al., 2017; Macaluso et al., 2021) or in prosthesis control (Holgate et al., 2009; Quintero et al., 2018; Hong et al., 2021); studies about the performance of the exoskeleton control methods based on them are more scarce, as will be discussed in detail in Section 2.1.2. For hip exoskeletons, the hip joint angle in the sagittal plane can be used as a basis for gait phase estimation. Thanks to its quasi-sinusoidal behavior in the gait cycle, a phase portrait plot (Strogatz, 2019), constructed from this signal and its derivative or integral, forms a closed curve with a semi-circular shape over each stride. The polar angle of this plot can therefore be a candidate phase variable. In a control strategy that we will refer to as Hip-Phase-based Torque profile controller (HPT) in this article, the %GC estimated from the polar angle of the phase portrait is used in conjunction with a predefined torque profile to determine the assistive torques.

Given the advantages and limitations of control strategies in each category of synchronization methods, selecting the appropriate controller for different applications is not trivial. Although many studies in the literature have explored the various control strategies and their performance in terms of metabolic cost reduction (Sawicki et al., 2020), these investigations have been conducted using different exoskeletons. Factors related to the exoskeleton hardware can heavily affect the performance of controllers, including the weight, mechanical structure, actuation method, body interfaces and the quality of the transfer of torques. Additionally, differences among protocols such as training time and walking conditions further complicate conclusive comparisons between the controllers. Comparative studies testing different controllers on the same device under similar conditions can therefore be helpful in disentangling the device- and experiment-related factors from the performance of the control strategy (Young and Ferris, 2017).

In this article, we investigate and compare the two aforementioned control strategies based on implicit and explicit synchronization. The first approach (SRC) uses implicit synchronization and continuously applies assistance torques, transitioning between hip extension assistance during stance and hip flexion assistance during swing. In contrast, the second controller (HPT) relies on a state-based explicit synchronization method and applies a predefined torque profile with two relatively short bursts of assistance in the extension and flexion directions. The timings of the bursts were inspired by the torque profiles obtained in previous studies using human-in-the-loop optimization for metabolic cost reduction. We chose these control strategies since they had potential advantages compared to other controllers within their respective synchronization category, but to the best of our knowledge, their performance had not been assessed in the existing literature. We mainly compared the performance of the two controllers in terms of their effects on metabolic energy expenditure in treadmill walking experiments. We initially hypothesized that in steady-state walking, the controller with explicit synchronization (the HPT) would yield higher metabolic reductions, thanks to a more targeted application of torques in terms of timing. Contrary to our expectations, however, the controller with implicit synchronization (the SRC) resulted in higher energy savings. We also explored the differences between the performance of each controller and the responses of the participants in terms of the kinematics of the hip joint, the applied torques, the assistive mechanical power and work, and the aggregate external work (performed by the human-exoskeleton system) on the center of mass.

## 2 Materials and methods

### 2.1 Control strategies

#### 2.1.1 Simple reflex controller (SRC)

**FIGURE 1:**
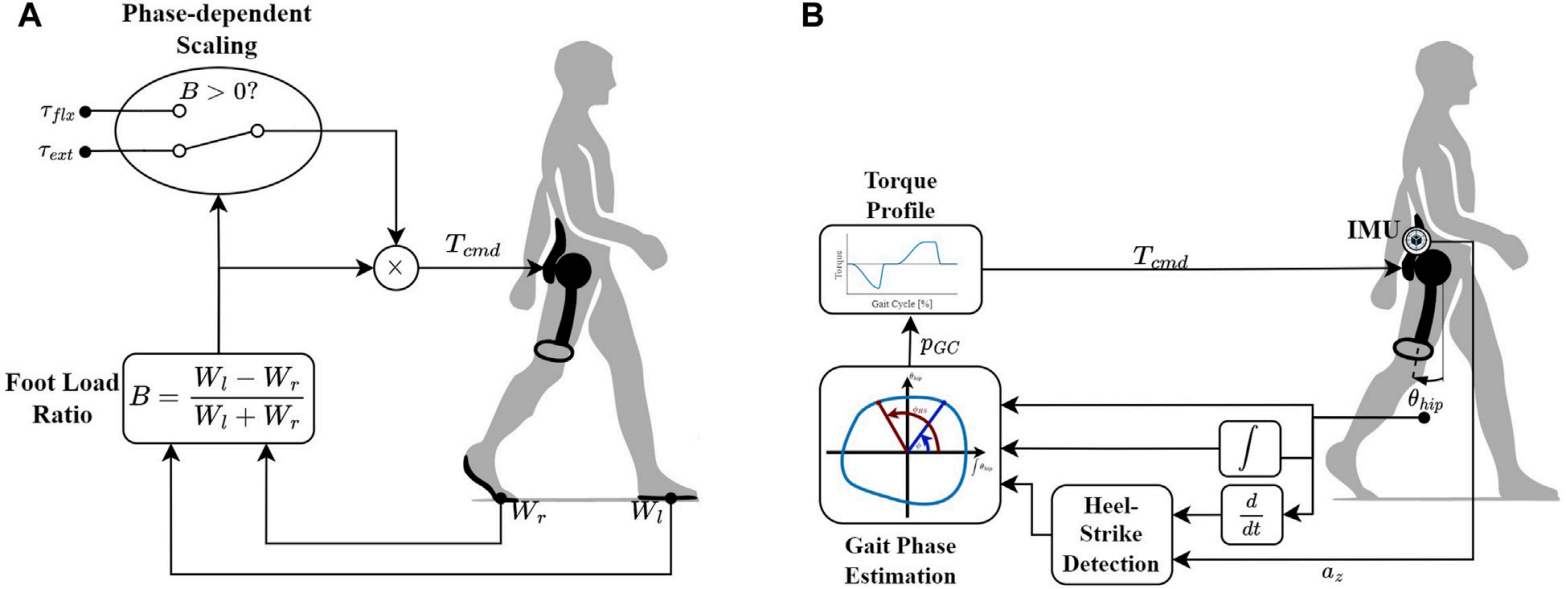
Block diagram representations of the two proposed controllers. **(A)** The Simple Reflex Controller (SRC) determines the torque command (*T*_*cmd*_) based on the dimensionless parameter *B*, which captures the difference between the weights borne by the left (*W*_*l*_) and right (*W*_*r*_) feet. As *B* changes sign between the phases, it is scaled by *τ*_*ext*_ during stance and by *τ*_*flx*_ during swing. **(B)** The Hip-Phase-based Torque profile controller (HPT) applies a predefined torque profile as a function of the gait phase (*p*_*GC*_) estimated from the phase portrait of the hip angle (*θ*_*hip*_). The offset added to *p*_*GC*_ is determined by the heel-strike detected using on a combination of the hip angular velocity and the vertical acceleration of the trunk (*a*_*z*_).

Reflexes are direct pathways from sensory signals to actions (Fisher, 2014), causing a rapid response to sensory inputs. Despite their simplicity, they can play a role even in complex behaviors such as gait, with varying degrees of importance among different animals and types of gait (Büschges, 2005; Duysens and Forner-Cordero, 2019). There even exist models that reproduce natural gaits solely using reflex-like mappings (Geyer and Herr, 2010), with combinations of reflexes that change as a function of gait phase. In spite of their useful features, the existing reflex-based controllers (also referred to as “Neuromuscular Controllers” (Tagliamonte et al., 2022)) have complex formulations and involve tens of parameters per joint that often need to be iteratively tuned. The computational ramifications and lengthy tuning procedures thus limit the usability of such approaches. The idea behind the SRC was therefore to use a simple structure, as illustrated in Figure 1A, to map a minimal set of sensory information to the appropriate assistive action.

**FIGURE 2:**
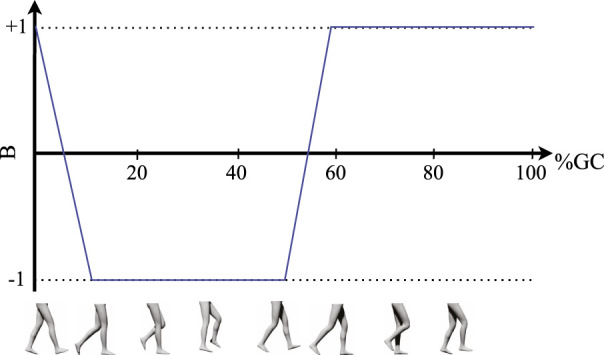
Schematic illustration of the typical evolution of the normalized GRF difference (function *B* defined in Eq. 1) over one full gait cycle, starting with right heel-strike.

Generally, the most fundamental set of gait phases used for modulating reflex pathways and the locomotor response in the reflex-based models is the stance/swing dyad. A natural choice for stance/swing differentiation is the initiation/termination of the loading of the legs, which has been shown to have a major role in the regulation of the reflexes and the neuro-muscular response during gait in different animals, including humans (Duysens and Pearson, 1980; Pearson, 1995; Duysens et al., 2000; Pearson, 2004). This information has also been utilized in simulations (Habu et al., 2018) and legged robot controllers (Maufroy et al., 2010; Macleod et al., 2014; Owaki and Ishiguro, 2017) to generate and modulate stable gaits. Leg loading is quantified based on the ground reaction forces (GRFs) on the feet. The GRF signals are straightforward to measure and easy to integrate into a hardware implementation in practice, using force/pressure sensors. Therefore, this signal was chosen as the only input to the controller. In order to map the GRFs to assistance torques in a simple yet robust manner, a memoryless and dimensionless function is proposed that captures the difference between the forces on each foot, as follows (Baud, 2020):

$$B\left(W_{l},W_{r}\right)=\frac{W_{l}-W_{r}}{W_{l}+W_{r}}$$ (1)

where *W*_*l*_ and *W*_*r*_ are the vertical GRFs on the left and right foot, respectively. The output of this function is +1 during the single-contact phase of the left leg, gradually transitions from +1 to −1 during the double-contact phase as the weight is transferred to the right leg, and retains the value of −1 during the entire single-contact phase of the right leg (Figure 2). The desired assistance torques for each hip joint are then defined by scaling the output of this function by the amplitude of flexion and extension assistance as:

$$T_{L}\left(B\right)=\left\{\begin{array}{cc} -B\;\tau_{\mathrm{ext}}\; & B>0\\ -B\;\tau_{\mathrm{flx}}\; & B\leq 0 \end{array}\right.$$ (2)

$$T_{R}\left(B\right)=\left\{\begin{array}{cc} B\;\tau_{\mathrm{ext}}\; & B<0\\ B\;\tau_{\mathrm{flx}}\; & B\geq 0 \end{array}\right.$$ (3)

where *T*_*L*_ and *T*_*R*_ are the assistance torques for the left and right hip joints, and *τ*_*ext*_ and *τ*_*flx*_ are the maximum values of assistance in the extension and flexion directions, respectively. It should be noted that in this study, torques in the extension direction are assumed to be negative, and those in the flexion direction are assumed to be positive. The only parameters that need to be tuned in this controller are *τ*_*ext*_ and *τ*_*flx*_. For our experiments, we set them to *τ*_*ext*_ = 135.0 mN ⋅ m ⋅ kg^−1^ and *τ*_*flx*_ = 67.5 mN ⋅ m ⋅ kg^−1^, both of which were subsequently scaled by the body mass of each user.

Note that due to the dimensionless nature of *B* in Eq 1, a precise measurement of the GRFs is not necessary. In this study, we used flexible force sensitive resistors (FSRs) in the insoles to estimate *W*_*l*_ and *W*_*r*_, without the need for an accurate calibration. Furthermore, these sensors did not measure the entire GRFs since they did not necessarily cover all of the contact points between the soles and the ground, depending on the size and shape of the feet. The normalization of the GRFs also makes the controller robust against asymmetries between the left and right GRF measurements.

In the final implementation, the desired torques given by Eqs 2, 3 were low-pass filtered in order to avoid discomfort due to the rapid transition between −*τ*_*ext*_ and *τ*_*flx*_, which occurs during the double-contact phase in around 100 ms. A first-order IIR filter was used for this purpose, with a cut-off frequency of *f*_*c*_ = 20 Hz which was found experimentally during pilot testing.

#### 2.1.2 Hip-Phase-based torque profile controller (HPT)

One of the methods for estimation of %GC without explicit reliance on time is to use a phase variable that encodes progression in the gait cycle (Villarreal and Gregg, 2014). Different candidate variables have been proposed and investigated in the literature, including the forward progression of center of pressure (for stance phase only) (Gregg and Sensinger, 2013; Gregg et al., 2014), horizontal position of the hip joint (Ames, 2012; Quintero et al., 2015), and the polar angles of the phase portrait (i.e., 2D plot of a state variable *versus* its time derivative) of different joint or segment angles (Villarreal and Gregg, 2014; Macaluso et al., 2021). For brevity, we will use the term “phase angle” to refer to the last one. The phase angle method has been more extensively studied, using the hip flexion/extension angle (Kerestes et al., 2014; Sugar et al., 2015), sagittal angle of the thigh (Bartlett and Goldfarb, 2018; Quintero et al., 2018) or shank segments (Holgate et al., 2009; Khazoom et al., 2019), virtual leg (the line connecting hip and ankle joints) angles (Sreenath et al., 2011; Ramezani et al., 2013; Villarreal and Gregg, 2014) or a linear combination of several joint/segment angles (Villarreal and Gregg, 2016) for building the phase portrait.

Despite being extensively used for control of prostheses and legged robots, very few studies have applied this method to exoskeletons. In an approach described by Kerestes et al. (2014) and Sugar et al. (2015), the phase of the hip flexion/extension angle is used for control of a hip exoskeleton, by triggering either extension or flexion assistance depending on whether the phase angle is above or below a threshold value. The idea behind this controller is to generate a periodic excitation signal that adds energy in phase with the cyclic movements of walking, but the details of their implementation are not described. Khazoom et al. (2019) have used the phase angle of the shank segment to parameterize the gait cycle for the control of an ankle exoskeleton, where the assistance profile is defined by directly mapping the phase angle to torques. The mapping was based on a predefined profile, which was manually tuned for each subject. Many of the parameters (such as the center point of the phase portrait and the phase angle corresponding to heel-strike and toe-off) were assumed to be constant, which limits the generalization of this approach to different walking conditions and persons.

In our implementation, we first use the phase angle of the hip flexion/extension angle to estimate the %GC. The assistive torques are subsequently determined as a function of the estimated %GC. (based on a predefined torque profile), as illustrated in the block diagram in Figure 1B. Similar to some previous studies (e.g., Villarreal and Gregg, 2016; Quintero et al., 2018; Hong et al., 2021), we used the hip angle and its first integral (rather than first derivative) to generate the phase portrait, in order to reduce the undesired effects of high-frequency noise and impact-induced local oscillations on the monotonicity of the phase angle. The procedure of constructing the phase portrait and calculating the phase angle has been described in detail in our previous work (Manzoori et al., 2023). Here, we only describe the central equation that captures the essential relationship between the phase angle and the %GC. The latter is estimated by calculating the normalized difference between the instantaneous phase angle and the phase angle at heel-strike:

$$p_{GC}=\left\{\begin{array}{cc} \frac{\phi_{HS}-\phi }{2\pi }\; & 0\leq \phi <\phi_{HS}\\ \;\\ \frac{2\pi +\left(\phi_{HS}-\phi \right)}{2\pi }\; & \phi_{HS}\leq \phi <2\pi \end{array}\right.$$ (4)

**FIGURE 3:**
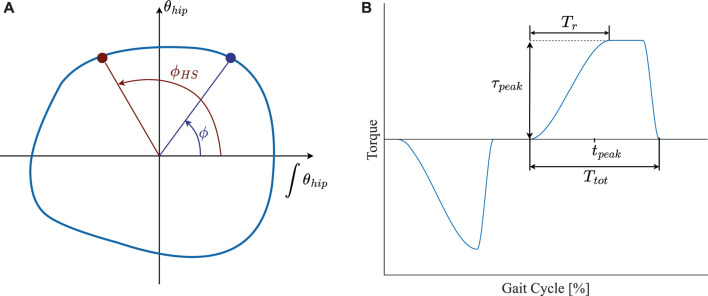
**(A)** Schematic illustration of the hip angle phase portrait (blue outline), showing the instantaneous (*ϕ*) and heel-strike (*ϕ*_*HS*_) phase angles. The phase portrait is obtained by plotting the hip angle (*θ*_*hip*_) against its time integral during walking. **(B)** The torque profile used in the Hip-Phase-based Torque profile controller (HPT) and its parameters: peak amplitude *τ*_*peak*_, peak time *t*_*peak*_, total duration *T*_*tot*_, and rise duration *T*_*r*_. The parameters are only shown for the flexion assistance bout, but the definitions are the same for the extension assistance bout.

Where *ϕ* and *ϕ*_*HS*_ are the instantaneous phase angle and phase angle at heel-strike (illustrated in Figure 3A), wrapped in the $\left.\left[0,2\pi \right.\right)$ range. Note that the radius of the phase portrait rotates clockwise and as a result, *ϕ* is decreasing over time; therefore, the first equation is used in the beginning of the gait cycle until the point where *ϕ* transitions from 0 to 2*π*. Thus, *p*_*GC*_ starts from 0 at the moment of heel-strike and continuously increases toward 1 at the ipsilateral heel-strike.

Contrary to most previous studies which reset the %GC to zero at a constant phase angle, in our approach the heel-strike event is detected in each gait cycle and used to reset the %GC. To keep the controller independent from foot load/contact sensing, heel-strikes were detected with an algorithm using a combination of the angular velocity of the hip and the acceleration of the trunk, as described in (Manzoori et al., 2023). The value of *ϕ*_*HS*_ is updated each time a new heel-strike is detected in the beginning of every cycle. The calculation of phase angle and %GC is carried out independently for the left and the right legs. To avoid issues due to local non-monotonic behavior of *ϕ*, in the software implementation, the value of *p*_*GC*_ is kept constant if it decreases compared to the previous sample, except for the moment of heel-strike.

The torque profile chosen in this study consists of one extension assistance and one flexion assistance peak, each having a semi-trapezoidal profile defined by four parameters: peak amplitude *τ*_*peak*_, peak time *t*_*peak*_ (timing of the middle of the peak), total duration *T*_*tot*_, and rise time *T*_*r*_ (Figure 3B). The fall time (i.e., duration of the decrease from the peak value back to zero at the end of the profile) was held constant at 5 %GC. The peak amplitude and time were set as *τ*_*peak*_ =135.0 mN ⋅ m ⋅ kg^−1^, *t*_*peak*_ = 10 %GC for the extension peak, and *τ*_*peak*_ =67.5 mN ⋅ m ⋅ kg^−1^, *t*_*peak*_ = 60 %GC for the flexion peak. The total duration and rise time were set as *T*_*tot*_ = 40 %GC, and *T*_*r*_ = 20 %GC for both of the peaks. We chose the values of peak time and total duration based on the profiles found in human-in-the-loop optimization studies (Franks et al., 2021; 2022). Peak amplitude and rise time, on the other hand, were found experimentally in pilot tests so as to prevent the discomfort of naïve subjects.

### 2.2 Hip exoskeleton

**FIGURE 4:**
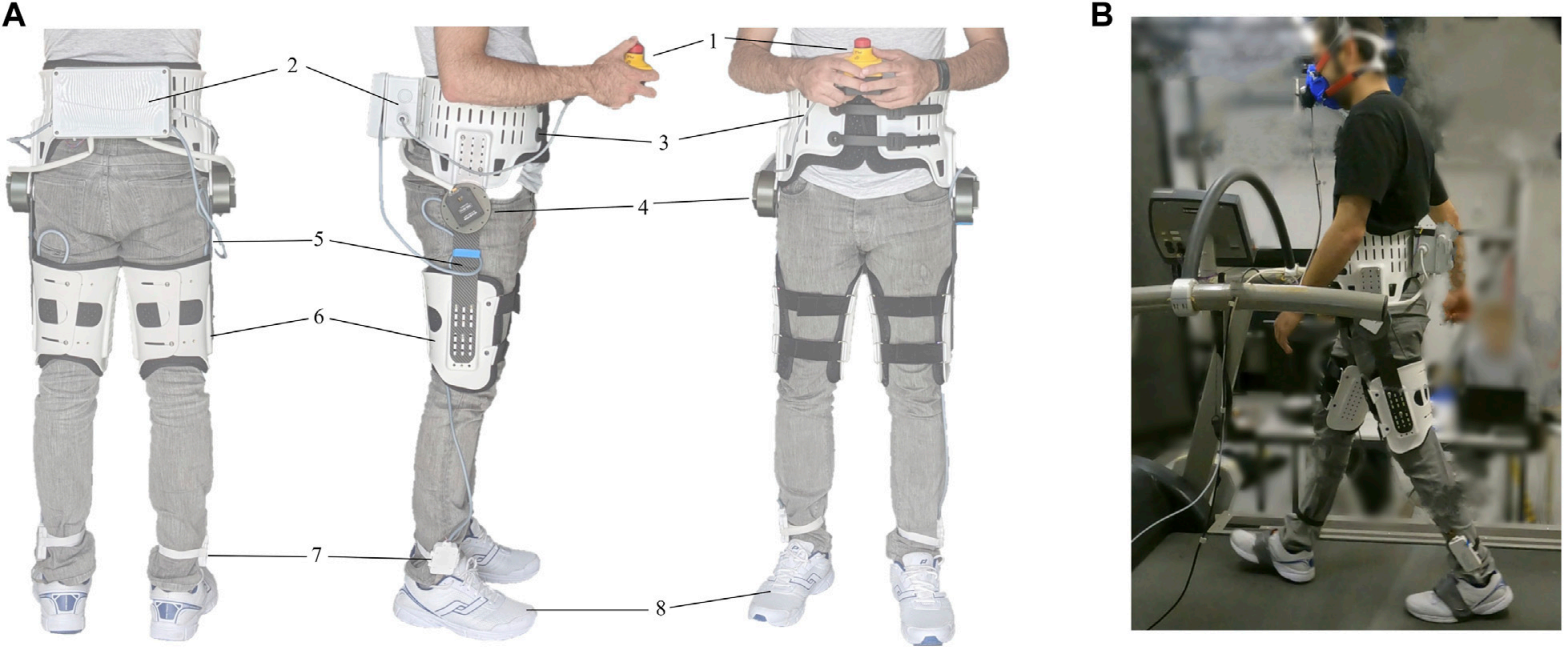
**(A)** The e-Walk V1 exoskeleton and its main components, numbered from top to bottom: (1) detachable emergency stop button, (2) computer and electronics enclosure, (3) waist attachment, (4) motors, (5) thigh segments, (6) thigh attachments, (7) foot sensor amplifier boards, (8) instrumented shoes. **(B)** The experimental setup showing a subject walking on the treadmill while wearing the exoskeleton and the gas exchange measurement mask.

An autonomous exoskeleton (e-Walk V1) developed for research was used in this study (Figure 4A). The exoskeleton attaches to the wearer’s waist and thighs using orthotic attachments made of flexible plastic lined with fabric. Two brushless DC motors mounted on the waist attachment actuate the hip joints in the sagittal plane. The motors have a 6:1 planetary reducer, providing nominal and peak torques of 13 and 35 N ⋅ m at the output, respectively. The efficient and low-ratio planetary reducers ensure the backdrivability of the motors, with an RMS back-driving torque of less than 0.6 N ⋅ m in cyclic movements with frequencies of up to 2 Hz. The motors are connected to the thigh attachments by thin rectangular segments made of carbon-fiber-reinforced polymer that are flexible around the sagittal axis. This flexibility facilitates passive freedom of the abduction/adduction movements in the small range required for normal walking. The exoskeleton is equipped with absolute joint angle encoders, motor current sensors, and an IMU (MPU-6050, InvenSense, United States) mounted near the lower-back of the wearer. Additionally, insole FSRs (8-cell Smart Foot Sensor, IEE, Luxembourg) with a minimum measurable force of 0.9 N are used to measure foot contact information. The exoskeleton lacks direct torque/force sensing, therefore the motor currents are used to estimate the applied torques, based on a linear relationship identified in bench-top calibration tests. The torque commands of the controllers were thus converted to current commands sent to the motor drivers running a closed-loop current control at 32 kHz. The commanded and measured torques have a resolution of 25 mN ⋅ m. The controller software runs on an embedded computer (BeagleBone Black, BeagleBoard.org Foundation, United States) at a frequency of 500 Hz. The exoskeleton is powered with lithium-polymer batteries, and the total weight of the device is 5 kg.

### 2.3 Experimental protocol and setup

#### 2.3.1 Participants

Twenty-three healthy participants (18 men and 5 women; age: 28 ± 9 years; body mass: 74 ± 8 kg; mean ± standard deviation) were recruited for this study. The protocol was reviewed and approved by the human research ethics committee of the canton of Vaud (CER-VD). All participants provided their written informed consent prior to participating in the experiments.

#### 2.3.2 Testing procedure

We tested the effectiveness of the assistance provided by the controllers in walking experiments at a moderate walking speed of 4 km ⋅ h^−1^ on an instrumented treadmill (T150-FMT-MED, Arsalis, Belgium). The experimental setup is shown in Figure 4B. On a first visit a few days prior to the main experiment, each participant walked on the treadmill for 5–10 min (depending on their level of experience with treadmill walking) while wearing the exoskeleton, starting in unassisted (transparent) mode and then gradually introducing assistance. The purpose was mainly to familiarize the participants with walking on the treadmill, and the duration was decided based on the results of Meyer et al. (2019).

During the experiment, four walking conditions were tested: without the exoskeleton (NO), with the exoskeleton in transparent mode (TR), assisted with the HPT controller (HPT), and assisted with the SRC controller (SRC). In the TR condition, the motors of the exoskeleton were commanded to apply zero torque. Each condition lasted 5 min to allow for the metabolic rate measurements to reach steady-state, followed by a rest period of 3 min. The order of conditions was pseudo-randomized. To avoid compounding possible short-term re-adaptations to the treadmill and the assistance (since the familiarization was done a few days before the experiment), the first condition was always unassisted (i.e., TR or NO).

We chose the parameters of the two controllers so as to have the same peak values for the assistance torques, i.e., 135.0 mN ⋅ m ⋅ kg^−1^ for extension and 67.5 mN ⋅ m ⋅ kg^−1^ for flexion assistance. Since the subjects were naïve, we chose relatively low values for the peak torques to prevent discomfort and reduce the need for adaptation, as evidence from past studies suggests that higher amplitudes of assistance necessitate longer adaptation times (Gordon and Ferris, 2007; Kao et al., 2010).

#### 2.3.3 Outcome measurements and processing

The data for some of the conditions had to be discarded due to issues with the exoskeleton or the measurements; one subject in TR and NO, four subjects in SRC, and five subjects in HPT were therefore excluded from the analysis.

##### 2.3.3.1 Metabolic rate of walking

To estimate the metabolic rate, expired gases (O_2_ uptake, $\dot{V}O_{2}$ , and CO_2_ output, $\dot{V}CO_{2}$ ) were collected using a metabolic cart (Quark CPET, Cosmed, Italy) on a breath-by-breath basis. The participants were asked to refrain from eating and drinking (except for water) from 3 h prior to the experiment, to limit the effect of the last meal before the experiment on indirect calorimetry to assess the metabolic rate. Volume and gas calibrations were performed before each trial. At the beginning of each session, a measurement was made in quiet standing for 5 min and averaged over the last minute to estimate the standing metabolic rate (W ⋅ kg^−1^) based on the energy equivalent of oxygen (Åstrand and Rodahl, 1977). Then, $\dot{V}O_{2}$ and $\dot{V}CO_{2}$ were measured during each walking condition with a respiratory exchange ratio of less than 1 for all participants and conditions. Breath-by-breath $\dot{V}O_{2}$ data were initially examined to exclude errant breaths due to coughing or swallowing, and values that were more than 3 standard deviations from the local mean were discarded. For each trial, $\dot{V}O_{2}$ values (mLO_2_ ⋅ kg^−1^ ⋅ min^−1^) from the last minute (i.e., steady state) were averaged and the gross metabolic rate (W ⋅ kg^−1^) was calculated using the same procedure as for the standing metabolic rate. Then, for all walking conditions, the standing metabolic rate was subtracted from the gross metabolic rate to calculate the net metabolic rate (W ⋅ kg^−1^).

##### 2.3.3.2 Hip joint kinematics and controller outputs

The flexion/extension angle and angular velocity of the hip joint, foot contact information, the parameters calculated by the controllers (i.e., the torque commands of both controllers and the estimated %GC by the HPT), and the applied torque by the exoskeleton were all logged by the embedded computer of the exoskeleton at 500 Hz. This information was not available for the NO condition, since the exoskeleton was not worn. The angles were measured by the motor encoders with a resolution of 0.0009◦ at the output of a gearbox with a backlash of 0.083◦, with the zero value occurring when the thigh and the trunk are aligned (for example, when standing straight). The angular velocity was calculated from numerical differentiation of the angle signal. During post-processing, both angle and angular velocity signals were low-pass filtered (zero-lag sixth-order Butterworth filter, cut-off frequency of 6 Hz). The torques applied by the exoskeleton were estimated from the motor currents. Angles, angular velocities, and torques in the direction of hip extension were taken to be negative, and positive in flexion.

We calculated the instantaneous power provided by the exoskeleton as the product of the applied torques and the angular velocities. We then numerically integrated the power profiles to obtain the exoskeleton work. We segmented the data into individual strides based on the offline detection of heel-strikes from insole FSR signals. Due to the symmetry of gait, we only used the values for the left leg in the analysis.

##### 2.3.3.3 Ground reaction forces, gait cadence, and individual limb power peaks

The total 3D GRFs were measured using the instrumented treadmill, equipped with four force plates with resolutions of 46 mN and 91 mN respectively in the horizontal and vertical directions. Force plate signals were sampled at 1 kHz. A validated algorithm (Meurisse et al., 2016; Bastien et al., 2019) was then used in post-processing to decompose the total force plate measurements into the individual limb GRFs during the last 30 s of each condition. We calculated the gait cadence based on the vertical component of the individual GRFs.

**FIGURE 5:**
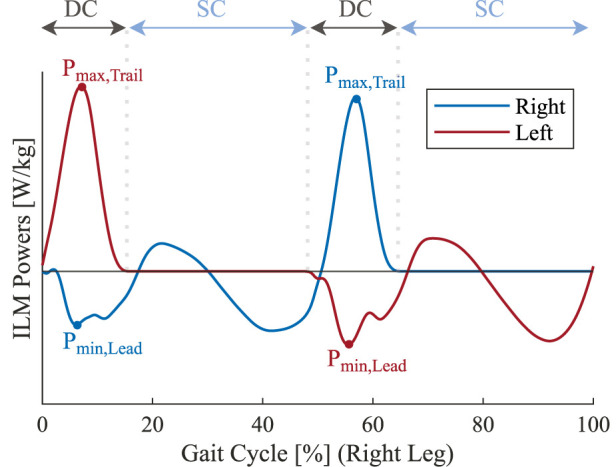
Schematic illustration of the individual-limb power profiles of each leg based on the individual limbs method (ILM) over one gait cycle starting with the heel-strike of the right foot. DC and SC labels denote the double-contact and single-contact phases, respectively. The maximum power of the trailing limb (*P*_*max, Trail*_) and the minimum power of the leading limb (*P*_*min, Lead*_) are marked over each double-contact phase.

We also used the individual GRFs to calculate the power exerted by each of the legs according to the individual limbs method (ILM) as described by Donelan et al. (2002b). After segmenting the data into single strides, we extracted the minimum and maximum values of the power profiles for the leading and trailing legs respectively (corresponding to weight acceptance and push-off) during the double-contact phase (illustrated in Figure 5), and averaged them over the gait cycles.

### 2.4 Statistical analysis

Statistical analysis was conducted using Jamovi software version 2.3.21.0 (The jamovi project, 2023). The different outcomes were compared among experimental walking conditions (fixed effect: NO vs. TR vs. SRC vs. HPT) with a linear mixed model, with participants set as a random effect to account for the repeated measures for each participant. The normality of the residuals was tested using the Kolmogorov-Smirnov test. The Holm correction was applied to identify where statistical differences in walking conditions (fixed effect) occurred. The level of significance was set to *p* ≤ 0.05. Correlation between metabolic rate responses to the two controllers was assessed using Pearson’s correlation coefficient in MATLAB version R2022b (The Mathworks Inc., United States).

## 3 Results

### 3.1 Net metabolic rate

**FIGURE 6:**
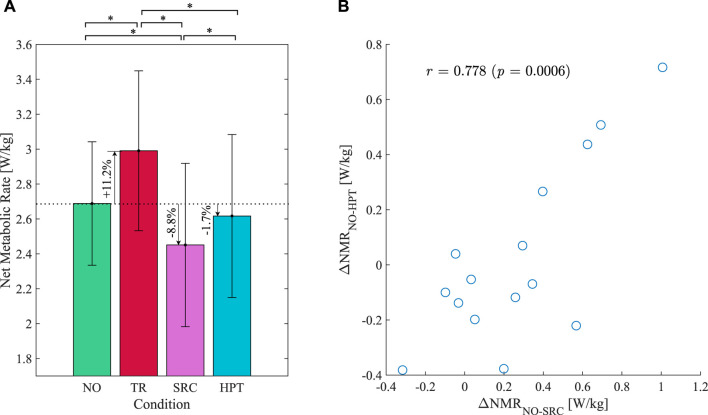
Results of the metabolic rate measurements. **(A)** Net metabolic rate of walking under the various conditions: without exoskeleton (NO), unassisted (TR), assisted with the Simple Reflex Controller (SRC), and assisted with the Hip Phase-based Torque profile controller (HPT). Error bars denote standard deviation. Asterisks denote pairwise significant difference with *p* <0.05. **(B)** Reductions in the net metabolic rate (ΔNMR) compared to the NO condition achieved with the SRC *versus* the HPT; the linear correlation coefficient and the corresponding *p*-value are also noted on the figure.

The net metabolic rate measurements in the different conditions are shown in Figure 6A. Wearing the exoskeleton without assistance (TR) led to a significant increase of 11.2% in the metabolic rate of walking compared to walking without the exoskeleton on average (*p* < 0.001). Assistance provided by the SRC significantly reduced the metabolic rate by 18.0% compared to TR (*p* < 0.001) and by 8.8% compared to NO (*p* = 0.004), on average. Assistance provided by the HPT significantly reduced the metabolic rate by 11.6% compared to TR (*p* < 0.001) and 1.7% (non-significant reduction) compared to NO (*p* = 0.487). Despite the considerable differences in the assistance provided by the two controllers and their corresponding metabolic benefits, a statistically significant strong correlation was observed between the net metabolic rate reductions (ΔNMR) obtained by each participant with the two controllers, as observed in Figure 6B. This indicates that the participants who had a relatively high metabolic rate reduction with one of the controllers often also benefited from a higher metabolic rate reduction with the other.

### 3.2 Exoskeleton torques, powers and works

**FIGURE 7:**
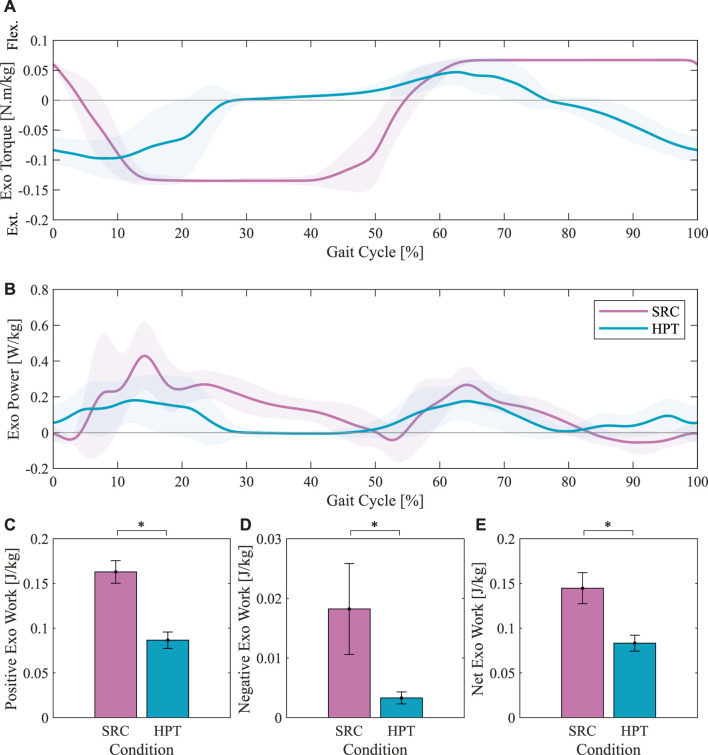
Exoskeleton torque profiles, power profiles, and works with the Simple Reflex Controller (SRC) and the Hip-Phase-based Torque profile controller (HPT). The average torque profiles are presented in **(A)**. The average power profiles over the gait cycle are illustrated in **(B)**. The average absolute values of the positive **(C)**, negative **(D)**, and net **(E)** works per stride are shown in the bottom panel. Asterisks denote pairwise significant difference with *p* <0.05.

The average assistance torque profiles applied by the two controllers are presented in Figure 7A. As expected, both profiles consisted of an extension assistance phase (negative sign) and a flexion assistance phase (positive sign). In the SRC profile, both phases had a longer duration compared to the HPT, each lasting around 50% of the gait cycle. In terms of timing, the HPT assistance onset preceded the movement in both phases, with extension and flexion assistance starting shortly before the beginning of the stance and swing phases, respectively. The SRC torques, on the other hand, lagged the movement and reached their peak value after the beginning of each phase. Note that although the peak values of the torques applied by the two controllers were equal, the peak torques in the averaged HPT profiles are lower due to the dispersion of the peak timings between different subjects. This inter-subject dispersion was due to the variability in the estimated %GC based on the phase portrait between different subjects. The peak values are more representative in the average profiles for individual participants, as shown in Supplementary Figure S1 for example. The SRC profiles showed less variability across different participants and strides, as evidenced by the smaller shaded area around the average profile in Figure 7A.

**FIGURE 8:**
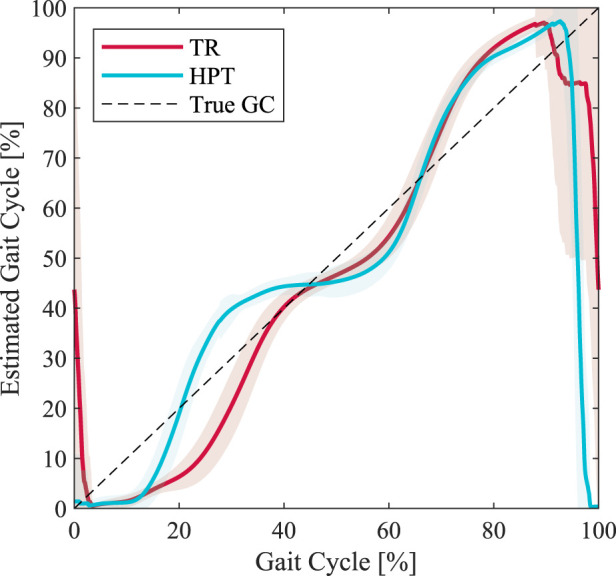
Estimated percent gait cycle (%GC) based on the phase portrait of the hip angle for a representative subject under the unassisted (TR) and assisted (HPT) conditions. The shown profiles are averaged over the entire 5-min duration. The true %GC is illustrated as a dashed line.

The HPT torque profiles were also slightly distorted compared to the ideal profile shapes (shown in Figure 3B). These deformations were due to the errors in the real-time %GC estimation, as observed in Figure 8. It can also be seen that the shape of the estimated %GC profile changes in the presence of assistive torques. This result is anticipated, since the assistance torques directly impacted the hip joint angle.

The average profiles of the mechanical power delivered by the exoskeleton with the two controllers are presented in Figure 7B. The average amounts of work per stride delivered by the exoskeleton are also shown in Figures 7C–E. The works and powers delivered by both controllers were predominantly positive, indicating that the torques applied by the exoskeleton were mostly aligned with the hip angular velocities. The SRC delivered significantly more work than the HPT (*p* < 0.001), in agreement with the contrast in the torque profiles. The difference was more remarkable in the negative work, where the SRC provided 5.5 times more negative work than the HPT (*p* < 0.001), while the ratio between the average positive works of SRC to HPT was around 1.9 (*p* < 0.001). The negative work performed by the SRC mostly occurred in late swing to early stance period and also in late stance, as can be seen in Figure 7B.

### 3.3 Hip joint kinematics and spatiotemporal parameters

**FIGURE 9:**
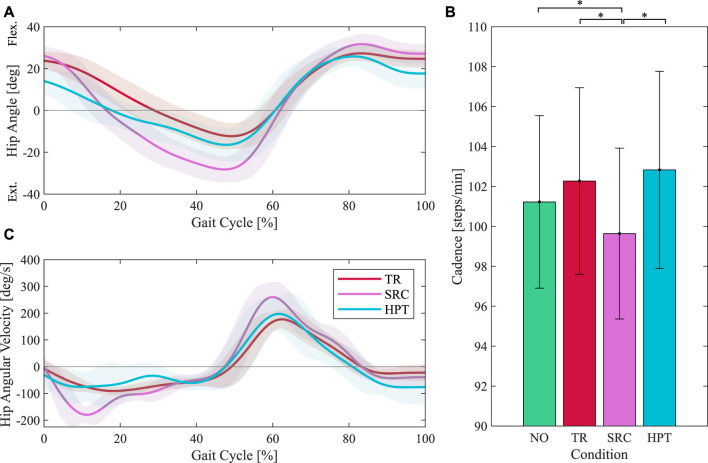
Hip joint kinematics and gait cadence in the unasssited (TR), assisted with the Simple Reflex Controller (SRC), and assisted with the Hip Phase-based Torque profile controller (HPT) conditions. The average hip angle and angular velocity profiles are shown in **(A, C)** respectively. The average values of cadence are displayed in **(B)**. Asterisks denote pairwise significant difference with *p* <0.05.

The hip joint angles and angular velocities measured by the exoskeleton are shown in Figures 9A, C. Compared to unassisted walking (TR), both controllers led to an increased range of motion, mostly in the direction of extension. This increase was more remarkable with the SRC, where the average peak extension angle is 12◦ more than in the TR condition. The angular velocity peaks are also visibly higher with the SRC, with two distinct peaks near early stance and early swing. The average cadence values are shown in Figure 9B. Compared to walking without the exoskeleton, participants slightly increased their cadence when walking in the unassisted mode, although this increase is not statistically significant (*p* = 0.179). Walking when assisted with the SRC led to a significant reduction in the cadence compared to all other conditions (*p*_SRC-NO_ = 0.017, *p*_SRC-TR_ < 0.001, *p*_SRC-HPT_ < 0.001), whereas the assistance provided by the HPT did not significantly alter the cadence compared to TR and NO (*p*_HPT-TR_ = 0.747, *p*_HPT-NO_ = 0.151).

### 3.4 Individual limb power peaks

**FIGURE 10:**
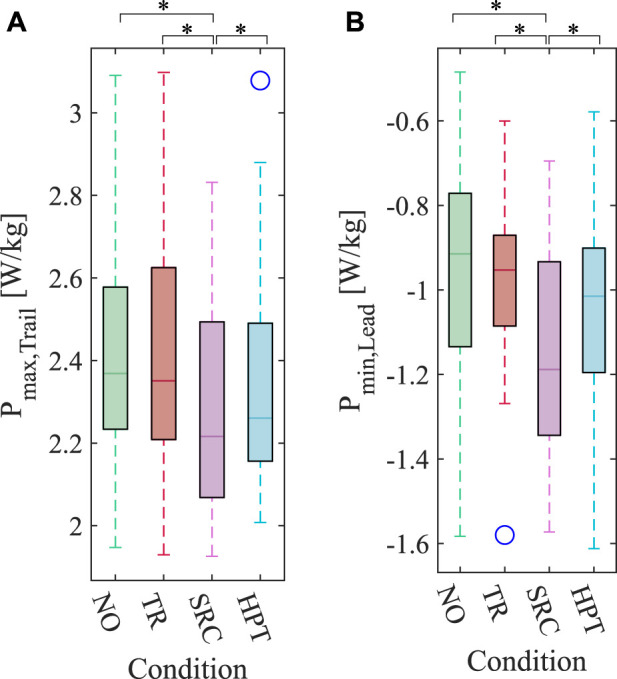
Box plot of the peaks of the power profiles for the trailing and leading legs calculated based on the individual limbs method (ILM), averaged over the last 30 s of each trial. **(A)** Peak of the positive (push-off) power applied by the trailing leg. **(B)** Peak of the negative power applied by the leading leg. The blue circles mark the outliers. Asterisks denote pairwise significant difference with *p* <0.05.

The average peaks of the power profiles applied by the trailing and leading limbs during double stance are presented in Figures 10A, B, respectively. For the trailing limb, the maximum power decreased in both assisted conditions. For the leading limb, on the other hand, the minimum powers increased in amplitude in the assisted conditions. In both cases, only the peak powers in the SRC condition were significantly different from the rest of the conditions (for the trailing limb: *p*_SRC-NO_ = 0.007, *p*_SRC-TR_ < 0.001, *p*_SRC-HPT_ = 0.044; for the leading limb: *p*_SRC-NO_ < 0.001, *p*_SRC-TR_ < 0.001, *p*_SRC-HPT_ = 0.004).

## 4 Discussion

In this work, our aim was to compare the performance of two control strategies for augmentative hip exoskeletons with key differences both in their design and in the provided assistance. In terms of design, the SRC directly maps sensory signals (i.e., relative foot loading information) to assistance torques at each instant and therefore, synchronization with the user is implicit. In contrast, the HPT operates based on explicit synchronization, estimating the %GC from the movement of the hip joint and applying torques according to a predefined profile as a function of the estimated %GC. As for the provided assistance, the SRC almost constantly applies torques with a fixed amplitude depending on the direction (flex./extension), whereas the HPT was designed to provide relatively shorter bursts of torque, but with the same amplitudes as the SRC. Our initial hypothesis was that the assistance provided by the HPT would result in greater metabolic savings when tested in steady-state treadmill walking, due to a more focused application of torques thanks to the predefined torque profile. Contrarily, the results showed that the SRC assistance led to a higher reduction in the walking metabolic rate. In the rest of this section, we will firstly discuss this outcome and the observed results in terms of energetics in more depth, followed by an assessment of the technical performance of each controller. Lastly, we will address the limitations of the study.

### 4.1 Effects of the controllers on metabolic rate

Both of the controllers could significantly reduce the metabolic rate during walking compared to the unassisted condition, but the reduction caused by the HPT was largely offset by the added metabolic cost of carrying the exoskeleton. The observed increase in the average metabolic rate as a result of wearing the exoskeleton without assistance (∼0.30 W ⋅ kg^−1^) is comparable to the value of 0.25 W ⋅ kg^−1^ predicted using the model proposed by Browning et al. (2007), with 4 and 1 kg of added mass at the waist and thighs, respectively^1^. However, this model was proposed for walking at 4.5 km ⋅ h^−1^, which is slightly faster than the speed used in our protocol, yet the added metabolic rate observed in our study is higher. This suggests that the added metabolic rate due to the exoskeleton extends beyond the impact of the added weight only. The extra increase is probably attributable in large part to the lack of a completely free abduction/adduction degree of freedom in the exoskeleton. Despite the flexibility of the thigh segments, the lack of a free hinge joint can lead to slight modifications of the step width, which are known to increase the metabolic cost of walking (Donelan et al., 2001).

Contrary to our hypothesis, the HPT did not yield greater metabolic benefits. The higher metabolic benefits obtained with the SRC can be explained by the higher duration of assistance (Figure 7A), which is also reflected in the higher mechanical power and work delivered by the exoskeleton with this controller (Figures 7C–E). Our initial reasoning was that, even though the SRC applies torques for a longer duration, the torque provided by the HPT would be more beneficial in the reduction of muscular effort due to better alignment in time with the biological torque profile of the hip joint. On the contrary, the participants seemed to benefit more from the prolonged application of torques by the SRC, despite the differences in timing compared to the biological torques. This result is similar to the findings of a previous study of hip assistance by Ding et al. (2016) in which assistive profiles with different timings and durations but similar amplitudes were compared, and the highest metabolic savings were obtained with the longest assistance duration.

Even though there was a positive correlation between the reductions in metabolic power and the delivered mechanical power/work by the exoskeleton, the metabolic rate reductions obtained by the two controllers were not proportional to the applied exoskeleton powers in this study. That is, while the average positive work applied by the exoskeleton was around 1.9 times higher with the SRC, the ratio between the average reductions in the metabolic power (with respect to TR) was only 1.5. This is also apparent in the graph illustrating the metabolic rate reductions versus the exoskeleton work for individual participants shown in Supplementary Figure S2. This observation further highlights the inherent limitation of indices such as the “augmentation factor” (Mooney et al., 2014) that assume direct proportionality between the reductions in metabolic power and the positive mechanical power provided by the exoskeleton.

The observed diminishing metabolic gain from the provided mechanical power by the exoskeleton could be due to several reasons. Firstly, a generally diminishing trend with increased assistance has been observed in some of the past studies with ankle exoskeletons (Jackson and Collins, 2015; Miller et al., 2022). It has also been observed in human-in-the-loop optimization studies that the best assistance strategies from a metabolic gain point of view are not those with the highest exoskeleton mechanical power (Zhang et al., 2017). The power delivered by the SRC might be less efficient in terms of reducing the effort, as it is not fine-tuned in terms of timing. Indeed, it can be observed that the torques applied by the SRC in some parts of the gait cycle are not biomechanically appropriate (i.e., extension torques in late stance and flexion torques in late swing to early stance). Lastly, it is also possible that the participants needed more training and experience to properly adapt to the assistance in order to fully leverage the higher applied powers.

In fact, in our protocol the participants had less than 5 min of initial exposure to each controller, only to mitigate immediate and rapid adaptation effects in the main trial. Since our primary goal was to compare the effects of two controllers under similar conditions, this aspect does not pose a substantial concern in this study. However, previous research suggests the importance of longer training times in fully exploring the potential of each assistance strategy for achieving metabolic benefits. Necessary training times for adaptation to assistance reported in prior research range from 10 min to 15 min (Lenzi et al., 2013), 24 min (Gordon and Ferris, 2007), to over an hour (Kao et al., 2010; Poggensee and Collins, 2021). Training over long periods of time (more than 4 h) has been shown to increase the metabolic benefits from the assistance by a factor of 3 compared to pre-adaptation levels (Poggensee and Collins, 2021). Therefore, the restricted training time in our study warrants cautious interpretation, as the attained metabolic benefits probably underestimate the full potential of the tested controllers.

We also assumed that the effect of short-term adaptation after less than 8 min of exposure to the assistance would be similar for the two controllers, since the levels of assistance (in terms of peak torque) were similar. However, we observed higher variability in the torques applied by the HPT, which is reflected in the higher standard deviation of the torques in Figure 7A. Therefore, it is also possible that this controller required longer adaptation times compared to the SRC, which applied a more consistent torque profile with little variation between strides.

As illustrated in Figure 6B, there was a significant correlation between the metabolic savings obtained by each subject while assisted by the two controllers. In other words, the participants who could benefit more from the assistance provided by one of the controllers were also more likely to gain more metabolic benefit when assisted by the other. We checked for potential confounding effects from age, weight, height, and body mass index but did not find any significant correlations. Since the assistance profiles provided by the two controllers were considerably different in terms of timing, duration, and applied mechanical power, this correlation may indicate a general difference between individuals in their capacity to utilize external assistance. This could be due to a difference in the rate of adaptation among the participants, meaning that with sufficient training time, the difference might wear off. Alternatively, the difference might be related to individual differences in gait pattern or long-term adaptability, making some individuals more predisposed to exploiting external assistance. The latter explanation is more likely, since individual differences in metabolic gain have been observed to be persistent after training in previous studies (Galle et al., 2013; Poggensee and Collins, 2021).

We tried to explore the possible biomechanical mechanisms behind the reduction in the metabolic rate by studying the external mechanical works on the center of mass using the ILM. Specifically, we focused on the double-stance phase, during which the external mechanical works may play a more pivotal role in influencing the metabolic rate (Donelan et al., 2002a). We found a statistically significant difference in the peak powers exerted by the trailing and leading limbs (*P*_*max, Trail*_ and *P*_*min, Lead*_) between the SRC and the rest of the conditions (Figure 10). In terms of magnitude, the trailing limb (which performs the positive work needed to accelerate the body forward) has a reduced peak power under the SRC condition; on the other hand, the leading limb (which performs the negative work needed to redirect the vertical movement of the center of mass) exhibits an increase in the peak power.

The reduced magnitude of *P*_*max, Trail*_ may indicate a reduction in the propulsion power provided by the ankle, which could be replaced by the extension assistance applied at the hip by the SRC during the entire stance phase. The increased acceleration as a result of this assistance on the trailing limb could also lead to a higher velocity of the center of mass during double-stance, bringing about the increase in *P*_*min, Lead*_ required to redirect the velocity. The values for the HPT also follow the same trend with respect to the NO and TR conditions, but the amplitude of the difference is not enough for statistical significance. This observation suggests that the obtained metabolic benefits may partly be due to reductions in the biological ankle push-off. This is in line with previous observations of reduced muscle activity or mechanical power in one joint as a result of assistance to other joints in both experimental studies (Franks et al., 2021; Jeong et al., 2023) and biomechanical simulations (Dembia et al., 2017).

### 4.2 Technical performance of the controllers

The mechanical power provided by the exoskeleton was predominantly positive under both controllers (Figure 7B). Since the applied torques were lower than the typical torques of the human joint for walking at normal speeds, the positive powers suggest that the assistance provided by both of the controllers were mostly aligned with the intended movements of the wearers. However, there are two visible periods of negative power provided by the SRC, one near late swing and early stance, and one during late stance. This is due to the reactive nature of this controller; the extension assistance only begins after the weight acceptance of the ipsilateral foot, and the flexion assistance starts after the foot is lifted. This can be clearly observed in the torque profile (Figure 7A), where the extension assistance (negative sign) starts around 5 %GC and the flexion assistance reaches its peak after 60 %GC. Conversely, the hip joint begins extending even before the heel-strike and flexing before the toe-off (Figure 9C).

Despite the short periods of negative power, the net effect of the assistance provided by the SRC was positive, as evidenced by the 18% reduction of the metabolic rate compared to walking with the passive exoskeleton. Furthermore, most of the participants preferred the assistance provided by the SRC, mentioning reasons such as “better synchronization” or “smoother assistance”. This is probably in part due to the torque profile which applies relatively long and continuous periods of assistance, as opposed to the shorter bursts of assistance applied by the HPT. Another reason could be the lower stride-to-stride variability of the SRC torque profile compared to the HPT, which can facilitate adapting to the assistance. The underlying reason for this lower variability is the fact that the SRC assistance is a function of the foot loads, which remain mostly invariant under assistance. The HPT, on the other hand, relies on the hip angle signal, which is directly affected by the applied torques, thereby creating a stronger interaction between the assistance and the wearer’s gait.

This interaction effect is clearly manifested in the difference between the unassisted and assisted conditions in the estimated %GC by the HPT, illustrated in Figure 8. For a few participants, this effect was occasionally resulting in high estimation errors that caused asynchronization of the torques with the wearer’s gait and severely altered it, which in turn led to even higher %GC estimation errors and eventually a complete malfunction of the controller. This occurred for four participants, who had to be excluded from the analysis. During the training period, we observed that this effect is more pronounced when the amplitude of the assistance is higher. Therefore, care should be taken in the design of controllers which use the state variables they are directly acting on, in order to avoid such unstable interaction loops.

As a result of their structural differences, each of the presented controllers have some advantages and limitations. The SRC proved to be more robust and produced a more repeatable assistance pattern, thanks to its simple design and implicit synchronization with the wearer. The inherent synchronization and the simple pattern of assistance also make this controller more adaptive to different types of gait. In preliminary tests, we have used this controller in slope and stair ascent successfully without any modifications to the parameters. But due to its reactive nature, the assistance has a bit of lag with respect to the movement of the joint, which leads to short periods of resistance to user’s movements. Also, fine-tuning the assistance profile is not straightforward as it emerges from the design of the controller. In contrast, the HPT allows for a detailed design of the assistance profile. However, the fidelity of the applied assistance in terms of timing is highly dependent on the accuracy of the %GC synchronization. Lastly, from a practical point of view, the HPT has the advantage of only requiring signals from the hip joint and the trunk, thereby reducing the complexity of added distal sensors located outside of the exoskeleton.

### 4.3 Limitations

There were some methodological limitations in this study which need to be addressed, mostly related to measurement constraints. Firstly, the joint angles and angular velocities were only measured by the encoders of the exoskeleton motors. However, these values can deviate from the actual hip joint angle due to the relative movement between the exoskeleton attachments and the body, as a result of imperfect fitting and soft tissue deformations caused by the applied torques. In addition, due to the absence of force/torque sensors in the exoskeleton, we used the electrical current of the motors to control and estimate the assistance torques. Even though we improved the accuracy of our estimations by conducting benchtop calibration tests with external torque sensors rather than directly relying on the torque constant of the motors, errors in the applied and measured torques remain inevitable (Yang et al., 2022). Also, no information was available about other joint angles and therefore we could not study the effect of the assistance on the full kinematics of the leg. Complementary studies with full-body/lower-body kinematic measurements using a separate motion capture system would provide more insight into the effects of these control strategies on users’ gait. Furthermore, including direct measurements of the exoskeleton torques would allow a more reliable application of the desired assistance profiles and more precise measurements of the resulting torques.

We only used metabolic rate as a measure of the overall effort, but it is not possible to accurately analyze the effects on the level of individual joints or muscles without muscle activity or joint torque information. We made speculations about the underlying physiological mechanisms behind the metabolic rate reduction by applying the ILM near the push-off period, when the ankle and knee joints are more dominant. Yet, our analysis was based on measurements from the hybrid human-exoskeleton system, and therefore the possibility of compound effects from the exoskeleton in our analysis cannot be completely ruled out. Including more microscopic and joint-specific measures of effort in the analysis would provide a richer perspective on the users’ response to each type of assistance.

Finally, in this work we only studied the performance of the controllers in steady-state treadmill walking. However, an important factor in the performance of assistive controllers is their robustness and adaptability in more real-world walking conditions where speed, cadence, and terrain are variable. Also, assistance can be more beneficial in more demanding tasks such as walking at faster speeds, on inclined ground, or while carrying loads. Expanding the scope of comparative experiments to encompass a wider range of walking conditions can therefore contribute to a more comprehensive understanding of the strengths and limitations of the controllers.

## 5 Conclusion

The SRC strategy (based on implicit synchronization) led to significantly higher metabolic benefits, with an average reduction of ∼9% compared to walking without the exoskeleton. This was against our hypothesis, which assumed the more targeted assistance of the HPT in terms of timing (thanks to explicit synchronization) to be more effective. The results demonstrated that the users could benefit from the added mechanical power provided by the SRC, despite the differences in timing compared to the biological power profiles. Indeed, we found some evidence indicating that the users might have modified the distribution of mechanical power among their joints and reduced their ankle push-off power when assisted with the SRC, in order to utilize the extra power. In addition, the fidelity of the torques applied by the HPT to the desired profile was limited by the online %GC estimation accuracy. These results were obtained in one session with very little previous exposure to the assistance, indicating that the controllers have the potential to achieve higher metabolic savings with more familiarization. Although the assistance profiles and the metabolic outcomes of the controllers were markedly different, a strong correlation was observed between the metabolic rate reductions achieved with the two controllers by each participant. This finding suggests differences in the general level of responsiveness to assistance (regardless of the torque profile and control strategy) among individuals, warranting further investigations.

## Data Availability

The raw data supporting the conclusion of this article will be made available by the authors, without undue reservation.

## Footnotes

1 We combined the regression models presented in Browning et al.‘s article for added mass to the waist and to the thighs into a single equation, namely, Net Metabolic Rate = 2.37 + 0.045 × *m*_*waist*_ + 0.075 × *m*_*thigh*_.
