# Supplementary material for: Evaluation of controllers for augmentative hip exoskeletons and their effects on metabolic cost of walking: explicit versus implicit synchronization
Source: Front Bioeng Biotechnol. 2024 Mar 12;12:1324587. doi: 10.3389/fbioe.2024.1324587 (PMC10963600; doi:10.3389/fbioe.2024.1324587)
Supplement: Supplementary file 1 [file DataSheet1.PDF]

Supplementary Material

1 SUPPLEMENTARY TABLES AND FIGURES

1.1 Figures

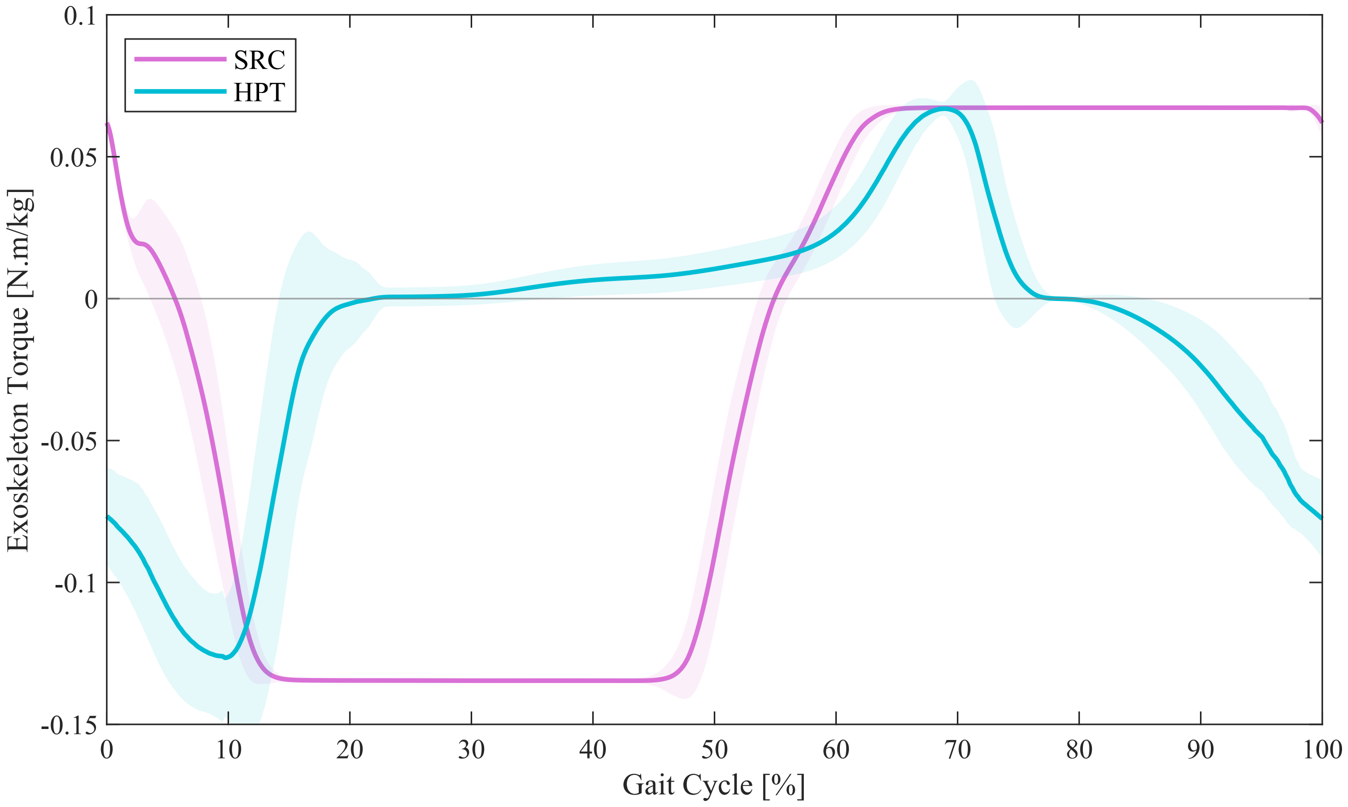

Figure S1. Average assistance torque profiles of the two controllers for a representative participant.

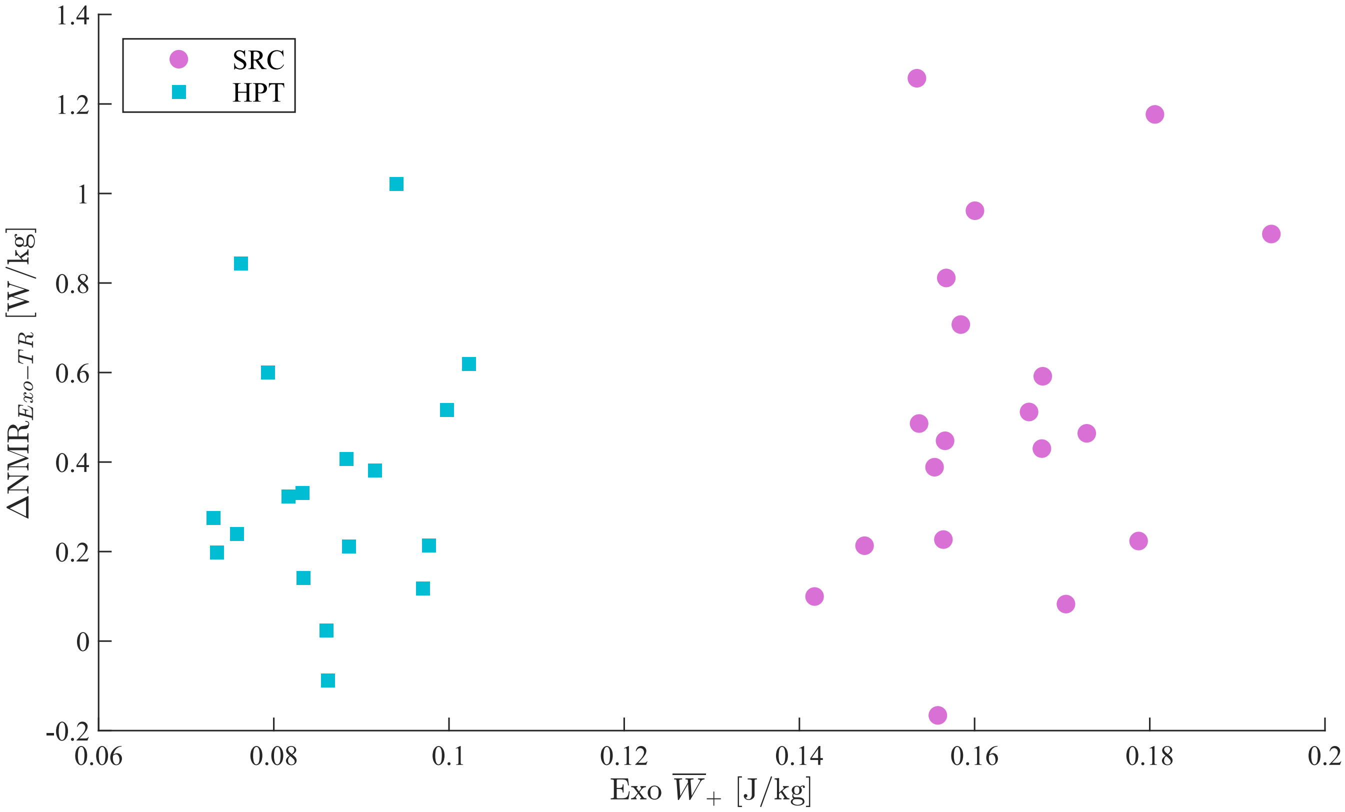

Figure S2. Net metabolic rate reduction versus average exoskeleton positive work for individual participants with the two controllers.
